# News

**Published:** 1915-02

**Authors:** 


### News.

In the last issue of the Journal we announced the appointment of Dr. E. D. Fyke of Fort Worth as State Health Officer. When his name was sent in to the Senate for confirmation a fight was made against him and he very graciously withdrew, his letter giving the reason for his resignation is as follows:

*Hon. James E. Ferguson, Governor of Texas, Austin, Texas.*

Dear Sir: Without solicitation on my part you tendered to me the appointment of State Health Officer. It now appears that my appointment to this important position is displeasing to many physicians throughout the State. The reason for such objection is immaterial. It is sufficient that it exists. Any man who hopes successfully to serve the public as Health Officer should have the friendly co-operation and sympathy of the physicians throughout the State. To me it is apparent that I cannot, at least at this time, procure that to the extent I deem it necessary.

I appreciate the high honor you have bestowed upon me. However, under existing conditions I deem it best to withdraw my acceptance of your tender of appointment, and ask you to accept this in the spirit in which it is made—an unselfish devotion to your administration and the public good.

Sincerely yours,

E. D. Fyke, M. D.

---

Honolulu, November 20, 1914.

*Texas Medical Journal, Austin, Texas.*

Dear Sirs: I was pleased to see a contribution in your last issue by our Dr. Goodhue. We are proud of him and of the fact that he has lately declined a flattering offer of partnership with a prominent practitioner in Los Angeles, where he was assured $10,000 a year for several years.

In a recent letter to me he says:

"I couldn't go, although in many respects we would advantage by the change. I was tempted, of course. But as I rode out one bright morning and felt the blessed Hawaiian air upon my face, breathed in the freshness of the mountain and sea, and realized that in going I must give up all that surrounds us here—friends not the least—I decided against going.

"I have flirted with opportunity many times, and declined a good many flattering positions to stay here, and have never been sorry.

"Yours,

